# Probing Dermal Immunity to Mycobacteria through a Controlled Human Infection Model

**DOI:** 10.4049/immunohorizons.2400053

**Published:** 2024-09-16

**Authors:** E. Chandler Church, Emma Bishop, Andrew Fiore-Gartland, Krystle K. Q. Yu, Ming Chang, Richard M. Jones, Justin K. Brache, Lamar Ballweber Fleming, Jolie M. Phan, Mohau S. Makatsa, Jack Heptinstall, Kelvin Chiong, One Dintwe, Anneta Naidoo, Valentin Voillet, Koshlan Mayer-Blackwell, Gift Nwanne, Erica Andersen-Nissen, Jay C. Vary, Georgia D. Tomaras, M. Juliana McElrath, David R. Sherman, Sean C. Murphy, James G. Kublin, Chetan Seshadri

**Affiliations:** * Vaccine and Infectious Disease Division, Fred Hutchinson Cancer Center, Seattle, WA; † Department of Medicine, University of Washington School of Medicine, Seattle, WA; ‡ Seattle-King County Public Health, Seattle, WA; § Department of Laboratory Medicine and Pathology, University of Washington School of Medicine, Seattle, WA; ¶ Department of Microbiology, University of Washington School of Medicine, Seattle, WA; ǁ Duke Center for Human Systems Immunology, Duke University, Durham, NC; # Cape Town HVTN Immunology Laboratory, Hutchinson Centre Research Institute of South Africa, Cape Town, South Africa; ** Department of Dermatology, University of Washington School of Medicine, Seattle, WA

## Abstract

Cutaneous mycobacterial infections cause substantial morbidity and are challenging to diagnose and treat. An improved understanding of the dermal immune response to mycobacteria may inspire new therapeutic approaches. We conducted a controlled human infection study with 10 participants who received 2 × 10^6^ CFUs of *Mycobacterium bovis* bacillus Calmette-Guérin (Tice strain) intradermally and were randomized to receive isoniazid or no treatment. Peripheral blood was collected at multiple time points for flow cytometry, bulk RNA sequencing (RNA-seq), and serum Ab assessments. Systemic immune responses were detected as early as 8 d postchallenge in this *M. bovis* bacillus Calmette-Guérin–naive population. Injection-site skin biopsies were performed at days 3 and 15 postchallenge and underwent immune profiling using mass cytometry and single-cell RNA-seq, as well as quantitative assessments of bacterial viability and burden. Molecular viability testing and standard culture results correlated well, although no differences were observed between treatment arms. Single-cell RNA-seq revealed various immune and nonimmune cell types in the skin, and communication between them was inferred by ligand-receptor gene expression. Day 3 communication was predominantly directed toward monocytes from keratinocyte, muscle, epithelial, and endothelial cells, largely via the migration inhibitory factor pathway and HLA-E–KLRK1 interaction. At day 15, communication was more balanced between cell types. These data reveal the potential role of nonimmune cells in the dermal immune response to mycobacteria and the utility of human challenge studies to augment our understanding of mycobacterial infections.

## Introduction

Cutaneous mycobacterial infections are caused by a variety of species and carry substantial morbidity. Chief among cutaneous mycobacterial infections are those caused by *Mycobacterium leprae*, the etiologic agent of leprosy, a disease for which the pathogenesis is poorly understood, treatments are difficult to tolerate and must be taken for an extended period of time, and that is associated with substantial disability even after cure (1). Similarly, *M. ulcerans* causes Buruli ulcer, a disease that is physically disfiguring and for which antibiotic treatment is limited to early disease (2). In high-income countries, cutaneous mycobacterial infections are caused by rapidly growing mycobacteria, such as *M. chelonae*, *M. gordonii*, and *M. marinum*, and are associated with soil or water exposure (3). Misdiagnosis of these infections is common, and they are often characterized by multidrug resistance (3). Cutaneous infection with *M. tuberculosis* or *M. bovis* is rare but does occur both as endogenous spread from pulmonary tuberculosis (TB) or exogenous infection as a result of contact with environmental bacteria (4).

The pathogenesis of cutaneous mycobacterial infection is poorly understood, which precludes the development of more effective treatments. Nontuberculous mycobacteria are rapidly cleared after injection into mice and do not reproduce the chronic infections seen in humans (5, 6). *M. leprae* cannot be cultivated in vitro and requires in vivo passage (7). Our limited understanding of the pathogenesis of leprosy comes primarily from detailed human studies. Leprosy occurs along a clinical spectrum, with multibacillary lepromatous leprosy at one extreme and paucibacillary tuberculoid leprosy at the other (8). These phenotypes are causally related to divergent T cell responses. Tuberculoid leprosy is characterized by a protective Th1 response, whereas lepromatous leprosy is characterized by a permissive Th2 response (9). However, other aspects of leprosy, such as the etiology of reversal reactions and erythema nodosum, as well as the mechanisms of nerve damage, continue to remain a mystery (10). A small study demonstrated that caseating necrosis was more prominent in patients with presentations consistent with TB verrucosa cutis and scrofuloderma, whereas patients whose lesions were more similar to lupus vulgaris had less necrosis (4). The CD4/CD8 ratio was also increased in lupus vulgaris compared with other forms of cutaneous *M. tuberculosis* (4). The pathogenesis of other cutaneous mycobacterial infections is even less well studied.

One approach to improving our understanding of cutaneous mycobacterial infections is to use controlled human infection models (CHIMs). Human challenge has not been previously conducted using live, fully virulent *M. tuberculosis* because of concerns about health risks to the participants and the potential for disease transmission to the community (11). By contrast, *Mycobacterium bovis* bacillus Calmette-Guérin (BCG) is the only vaccine currently available against *M. tuberculosis* and is given to millions of infants and children worldwide each year via the intradermal route. Because it is a live attenuated vaccine, BCG has been studied as a surrogate for *M. tuberculosis* in CHIMs. Intradermal challenge with BCG in both BCG-naive and -experienced participants against a candidate TB vaccine, MVA85A, showed lower amounts of BCG bacteria at 2 wk in BCG-experienced participants, but no difference by MVA85A status, consistent with the later trial results for MVA85A (12, 13). Mycobacterial shedding after BCG skin challenge was also shown to correlate with several potential systemic biomarkers of mycobacterial immunity (14). Finally, BCG can be safely inoculated into the lungs of humans at high doses via bronchoscopy with resulting immune responses localized to the inoculated lung compartment (15). Recent work has shown that both aerosol and intradermal BCG were generally well tolerated in a dose-escalation study that also demonstrated recovery of BCG from bronchoscopy samples taken 2 wk after aerosol challenge (16). In all these studies, standard microbiologic approaches were used to measure bacterial burden.

Assessing bacterial growth on solid or liquid media remains the gold standard for quantitative analysis of mycobacterial burden but is laborious and takes several weeks to obtain a result. In contrast, molecular approaches such as PCR provide more rapid results but cannot reliably distinguish viable from nonviable bacteria (17). We used a new assay, referred to in this article as the molecular viability test (MVT), to detect only actively dividing BCG given the short half-life of pre-rRNA (18–21). Previously, we demonstrated that this MVT, which uses RT-PCR to quantitate the pre-rRNA/rDNA peak ratio to measure the amount of BCG before and after isoniazid (INH), correlated well with culture positivity in an intradermal mouse model (20).

Although CHIMs have been targeted primarily toward vaccine development, there is also potential for use in drug development and evaluation of host-directed therapies (22). We designed this phase I clinical trial to investigate the potential role of MVT ratio in distinguishing between drug-treated and untreated participants in the context of BCG CHIM. We also performed serial biopsies and comprehensively characterized the dermal immune response to BCG in both groups of participants. The local immunology of BCG in the skin, particularly in the presence of treatment, has not been extensively examined in prior CHIM studies. Our study establishes the utility of MVT in the context of CHIM and reveals new insights into the role of nonimmune cells in the dermal immune response to mycobacterial infection.

## Materials and Methods

### Study design

This was a randomized, open-label trial investigating the local and systemic immune response to BCG and the impact of INH on detectable mycobacteria in skin biopsies. The study enrolled 10 BCG-naive adults aged 18–45 y who were judged to be in general good health as determined by medical history, physical examination, and laboratory tests. All participants assigned female sex at birth agreed to consistent use of contraception; pregnant or breastfeeding persons were excluded (Supplemental Table I). All participants were enrolled at a single site in Seattle, Washington. The trial was registered at ClinicalTrials.gov (NCT05592223). Participants received 0.1 ml of BCG (Tice strain) reconstituted with 20 ml of saline, a dose estimated to equal 2 × 10^6^ CFUs. Participants were randomized one to one to receive INH 300 mg daily for 3 d or no treatment. The dose and timing of INH were selected based on our preclinical studies demonstrating a substantial reduction in BCG burden in mice that received 2 × 10^6^ CFUs BCG followed by 4 d of INH (20). Randomization was performed using random number generators. Given the small sample size, once one arm of the study was filled, all remaining participants were automatically assigned to fill the remaining arm. Participants were followed for 114 d postvaccination. Adverse events (AEs) were collected throughout the study via a daily electronic survey sent through RedCap.

### Ethics

The study was approved by the Fred Hutchinson Cancer Center Institutional Review Board (Institutional Review Board no. 10903).

### Skin biopsy processing

On each of days 3 and 15 post-BCG vaccination, one 4-mm skin biopsy (Thermo Fisher Scientific) was collected from the forearm of each participant, with the exception of one participant who declined a day 15 biopsy (non-INH treated). The biopsy was dissected in half, and each half was immediately placed into a 1.8-ml cryovial with 1 ml of 1× PBS (Life Technologies). One set of the biopsies was processed for microbiology assays, including MVT and mycobacterial culture, and the other set was used for immunology assays, including mass cytometry and single-cell RNA sequencing (scRNA-seq). All laboratory assays were performed blinded to treatment group. We could not generate single-cell sequencing data from five biopsies because of low cell yield or missed collection (one from day 3 INH group and four from day 15 non-INH group).

Samples to be used for microbiology assays were removed from PBS and placed into 2 ml of Mycobacterial Growth Indicator Tube (MGIT) media. These were placed on a gentleMACS dissociator (Miltenyi Biotech) and ground using the “M tube protein_01” protocol according to manufacturer’s instructions. Samples were then centrifuged to separate tissue debris from the supernatant. From the centrifuged tubes, 0.6 ml of supernatant was taken to prepare dilutions for plating on solid media. This supernatant was serially diluted to 10^−5^, and all dilutions were plated in duplicate on Difco Middlebrook 7H10 Agar (VWR) with and without the addition of polymyxin B, amphotericin B, nalidixic acid, trimethoprim, and azlocillin (BD Biosciences) for quantitative bacterial assessments. The plates were incubated at 37°C for 28 d, and the number of CFUs was determined. CFU values were doubled to approximate the CFUs present in the entire punch biopsy. An additional 0.4 ml of supernatant was transferred to two vials of NucliSENS lysis buffer (Biomérieux) and stored at −80°C for baseline MVT assessment. In addition, 0.3 ml of supernatant was added to additional MGIT media and incubated at 37°C. The culture was sampled at days 0, 2, 3, 4, and 5 and transferred into lysis buffer before being cryopreserved at −80°C for additional MVT assessments.

Samples to be used for immunology assays were placed into a 10-cm sterile petri dish, and the tissues were further dissected into three to four smaller pieces using sterile scalpels and forceps. For each participant, the tissues were then placed into GentleMACS C tubes (Miltenyi Biotec), containing Enzymes D, A, and P prepared according to the manufacturer’s protocol for the Whole Skin Dissociation Kit (catalog no. 130-101-540). The samples were incubated overnight in a 37°C water bath. The following day, the samples were processed according to the manufacturer’s protocol. In brief, the samples were diluted by adding cold medium consisting of RPMI 1640 (Life Technologies) supplemented with 10% FBS (Hyclone), 0.3X essential amino acids, 60 mM nonessential amino acids, 11 mM HEPES, 800 mM l-glutamine (Life Technologies), and 50 mg/ml carbenicillin (Thermo Fisher Scientific), sterile filtered, and then dissociated using the gentleMACS dissociator and the C tube protocol h_skin_01. The samples were then briefly centrifuged at 300 × *g* for 30 s and filtered through a 35-μm cell strainer (Thermo Fisher Scientific). After washing twice with cold cell culture medium and centrifuging at 300 × *g* for 10 min, the samples were resuspended in 1 ml of FACS Buffer (1× PBS [Life Technologies] supplemented with sterile filtered 0.2% BSA [Sigma]). Viable cells were enumerated using the Guava easyCyte (MilliporeSigma) with guavaSoft 2.6 software and then resuspended as needed for the mass cytometry and scRNA-seq assays.

### Molecular viability testing

Cryopreserved samples from days 0, 2, 3, 4, and 5 of MGIT liquid culture were thawed and vortexed. The NucliSENS easyMag instrument was used to extract DNA and RNA. Samples were run in duplicate for BCG RT-PCR and PCR. Primers and probe sequences were used as recently published (20). The ratio of pre-rRNA to rDNA was 2, powered by the absolute value of the difference between cycle numbers from RT-PCR and PCR (2^[|(CNRTPCR-CNPCR)|]^) and calculated for all time points. Any ratio > 1 was considered “positive,” indicating the presence of live BCG in the sample. Samples positive for pre-rRNA but negative for rDNA were also considered positive ratios but were not calculated for these samples. The primers used were as follows: probe, 5′-6FAM-TTTGATCCTGGCTCAGGACGAACG-3′; forward primer, 5′-TCTAAATACCTTTGGCTCCCTTT-3′; and reverse primer, 5′-CGTTCGACTTGCATGTGTTAAG-3′ (20).

### Binding Ab multiplex assay

Humoral immune responses were assessed using a flow cytometry–based technology that uses Ab and Ag interactions to test for the simultaneous presence of multiple specific Abs in a serum sample, as previously published (23). Partially purified *M. tuberculosis* Ag and BCG Tice suspension are coupled to uniquely assigned fluorescent bead sets that are then mixed to allow for multiplexing. The *M. tuberculosis* Ags used are *M. tuberculosis* whole-cell lysate (WCL; strain H37Rv; BEI Resources) and *M. tuberculosis* lipoarabinomannan (LAM) (strain H37Rv; BEI Resources). Serum samples are serially diluted at a starting 1:50 dilution in assay diluent and added to the multiplexed bead sets within a 96-well plate; samples and controls are tested in duplicate. Serum sample and bead sets are mixed for an incubation period of 30 ± 5 min at 22 ± 2°C. After sample incubation, bead sets are washed with assay wash buffer. The wash step is followed by the addition of a polyclonal goat anti-Human IgG Fc secondary Ab conjugated to PE to allow for a measurable fluorescent readout. Samples and controls are mixed with the secondary Ab detection for a 30 ± 5 min incubation period at 22 ± 2°C. Before reading the 96-well assay plate, bead sets are washed with assay wash buffer to remove any unbound material. Bead sets are acquired and read using a Bio-Rad 200 system with BioPlex manager operating software (version 6.1.1). The readout is background-subtracted mean fluorescence intensity, where background referred to a plate-level control (i.e., a blank well run on each plate). Standard positive and negative controls are included in each assay to ensure specificity and to maintain consistency and reproducibility between assays. The positive controls include titrated anti–*M. tuberculosis* LAM mAb and anti-Ag85 polyclonal Ab standards in addition to known human plasma positive for *M. tuberculosis* Abs. The negative controls include normal human sera and blank beads.

### Intracellular cytokine staining

Intracellular cytokine staining (ICS) was performed on cryopreserved PBMCs as previously described (24). In brief, PBMCs were thawed in R10 media RPMI 1640 (Life Technologies) supplemented with 10% FBS (Nucleus Biologics), 2 mM l-glutamine, 100 U/ml penicillin G, and 100 mg/ml streptomycin sulfate (Life Technologies), counted on a Muse Cell Analyzer (Cytek Biosciences), and incubated overnight at 37°C/5% CO_2_. The following day, PBMCs were plated at 1.2 million cells/well and then stimulated for 8 h at 37°C/5% CO_2_.

For BCG Tice stimulations, vaccine vials containing 1–8 × 10^8^ CFUs (Merck & Co.) were reconstituted in RPMI and used to stimulate cells, using an estimate of 5 × 10^8^ CFUs per vaccine vial. A titration of BCG Tice was performed to determine the optimal concentration for simulation, testing multiplicities of infection of 1–16 relative to an estimated proportion of monocytes of 10% in the PBMCs. Gamma-irradiated *M. tuberculosis* H37Rv WCL (catalog no. NR-14822; BEI Resources) was used at a final concentration of 30 μg/ml (25). Staphylococcal enterotoxin B (MilliporeSigma) at a final concentration of 0.25 mg/ml was used as a positive control, and PBS (Life Technologies) was used as a negative control. Stimulation cocktails were prepared in PBS in the presence of costimulatory Abs CD28 and CD49d (BD Biosciences) at 1 µg/ml. Brefeldin A (MilliporeSigma) was added at 10 µg/ml, 2 h after the start of the stimulation. Six hours later, at the end of the total stimulation time of 8 h, EDTA (Life Technologies) was added at a final concentration of 2 mM, and the samples were stored overnight at 4°C. The following day, the cells were stained with a 28-color panel: LIVE/Dead Fixable Blue (Invitrogen), followed by MR1 5-OP-RU tetramer Ax488 (National Institutes of Health [NIH] Tetramer Core Facility), anti-TNF BUV395 (clone Mab11; BD Biosciences), anti-CD45RA BUV496 (clone HI100; BD Biosciences), anti-TCR Vα24-Jα18 BUV563 (clone 6B11; BD Biosciences), anti-TCR γδ BUV661 (clone 11F2; BD Biosciences), anti-CD154 (CD40L) BUV737 (clone TRAP1; BD Biosciences), anti-CD8α BUV805 (clone SK1; BD Biosciences), anti–IFN-γ V450 (clone B27; BD Biosciences), anti-CD4 BV480 (clone SK3; BD Biosciences), anti-NKG2C BV650 (clone 134591; BD Biosciences), anti-CCR6 BV711 (clone 11A9; BD Biosciences), anti–IL-4 BB630 (clone MP4-25D2; BD Biosciences), anti–IL-13 BB630 (clone JES10-5A2; BD Biosciences), anti-Ki67 BB660 (clone B56; BD Biosciences), anti–IL-2 BB700 (clone MQ1-17H12; BD Biosciences), anti-CD14 BB790 (clone M0P9; BD Biosciences), anti-CXCR3 PE-Cy5 (clone 1C6/CXCR3; BD Biosciences), anti-CD16 BV570 (clone 3G8; BioLegend), anti-TCR Vδ2 BV605 (clone B6; BioLegend), anti-CD56 BV750 (clone 5.1H11; BioLegend), anti-CCR7 BV785 (clone G043H7; BioLegend), anti-Granulysin PE (clone DH2; BioLegend), anti–GM-CSF PE-CF594 (clone BVD2-21C11; BioLegend), anti–IL-17a PE-Cy7 (clone BL168; BioLegend), anti–HLA-DR PE-Cy5.5 (clone TU36; Thermo Fisher Scientific), anti–IL-17F PE-Cy7 (clone SHLR17; Invitrogen), anti-CD153 allophycocyanin (clone 116614; R&D Systems), and anti-CD3 allophycocyanin-Fire 750 (clone UCHT1; BioLegend). Data were acquired on a BD FACSymphony A5 flow cytometer (BD Biosciences) equipped with UV (355 nm), violet (405 nm), blue (488 nm), green (532 nm), and red (628 nm) lasers.

Data were gated and analyzed using FlowJo version 9.9.4 (FlowJo) (Supplemental Fig. 2). COMbinatorial Polyfunctionality analysis of Ag-Specific T cell Subsets (COMPASS) (v1.13.0) was used to compute polyfunctionality scores (PFSs) (26). COMPASS uses a Bayesian framework to identify T cell subsets for which there is a high likelihood of an Ag-specific response. It compares the proportion of responsive cells in stimulated samples against control samples. The COMPASS PFS summarizes the overall functional profile of a subject’s T cells into a single number for further analysis.

### Whole blood mRNA sequencing

RNA isolation from whole blood cryopreserved in Tempus blood RNA Tubes (Thermo Fisher Scientific) was performed according to the manufacturer’s instructions using the Tempus spin RNA Isolation kit (Thermo Fisher Scientific). Globin depletion from total RNA from whole blood was performed according to the manufacturer’s instructions using the GLOBINclear-Human Kit (Thermo Fisher Scientific). Stranded poly-adenylated mRNA-seq libraries were generated from 50 ng of globin-depleted whole blood RNA using the Advanta RNA-seq XT NGS library prep kit with unique dual indices (Standard Biotools) using the 48-Atlas Integrated Fluidic chip on the Juno Instrument (Standard Biotools) according to the manufacturer’s instructions and sequenced on the Illumina NovaSeq 6000 sequencing platform (Illumina).

Samples had a median of 23 million reads mapped to protein-coding genes with a minimum of 5 million reads. Reads were processed using the in-house *nf-core/rnaseq* pipeline, with gene-level quantification performed using salmon (27, 28). Differentially expressed genes (DEGs) were identified using linear mixed-effects models of log CPMs, with each day compared with baseline day 0 and using participant ID as the grouping variable. Models were fit for each gene using the R package kimma (29). Significant DEGs had a nominal *p* < 0.05, false discovery rate (FDR) adjusted q value < 0.2, and absolute log_2_ fold change (log_2_FC) > 0.5.

Gene set enrichment analysis was conducted by looking for DEGs that were overrepresented in previously established vaccine-related gene sets from Li et al. (30). To compute enrichment scores, we used odds ratios and a hypergeometric test from the *enricher* function from the clusterProfiler package (31), with all detected genes as the gene “universe.” The 346 blood transcriptional modules (BTMs) were used as gene sets (30). Gene sets with enrichment of upregulated or downregulated DEGs from each time point were significant if the FDR adjusted q value was <0.05.

### Multimodal scRNA-seq

Each donor’s sample was centrifuged and resuspended in 100 ml of FACS buffer and then assigned to 1 of 10 TotalSeq-C Hashtag Abs (BioLegend). Samples were incubated for 30 min at 4°C and then washed twice with 500 ml of FACS buffer with centrifugation at 700 × *g* for 3 min. A total of 20,000 viable cells from each sample were pooled and then blocked with an Fc Receptor Binding Inhibitor (Invitrogen) for 10 min at 4°C prepared 1:10 in FACS buffer. The pooled sample was then centrifuged at 700 × *g* for 3 min and then stained with a TotalSeq-C (BioLegend) cellular indexing of transcriptomes and epitopes by sequencing (CITE-seq) panel containing 39 markers: anti-CD3 (clone UCHT1), anti-CD4 (clone RPA-T4), anti-CD8α (clone RPA-T8), anti-TCR γδ (clone B1), anti-TCR Vα7.2 (clone 3C10), anti-CD62L (clone DREG-56), anti-CCR7 (clone G043H7), anti-CD45RA (clone HI100), anti-CD28 (clone CD28.2), anti-CD127 (clone A019D5), anti-CD95 (clone DX2), anti-CXCR3 (clone G025H7), anti-CXCR5 (clone J252D4), anti-CCR4 (clone L291H4), anti-CCR5 (clone J418F1), anti-CCR6 (clone G034E3), anti-CD25 (clone BC96), anti-CD38 (clone HIT2), anti-CD26 (clone BA5b), anti–HLA-DR (clone L243), anti-CD161 (clone HP-3G10), anti-CD69 (clone FN50), anti-CD103 (clone Ber-ACT8), anti-CD14 (clone M5E2), anti-CD16 (clone 3G8), anti-CD56 (5.1H11), anti-CD11b (clone ICRF44), anti-CD11c (clone S-HCL-3), anti-CD169 (clone 7-239), anti–PD-L1 (clone 29E.2A3), anti-CD19 (clone HIB19), anti-CD123 (clone 6H6), anti-CD15 (clone W6D3), anti-CD20 (clone 2H7), anti-CD163 (clone GHI/61), anti-CD86 (clone IT2.2), and IgG2a, IgG1, and IgG2b isotype controls. After a 30-min incubation at 4°C, the sample was washed twice with 200 ml of FACS buffer, and then viable cells were enumerated using the Guava easyCyte. Cells were filtered through a cell strainer, and 33,000 pooled cells per well were prepared for loading into each of two Chromium Next GEM Chips (10× Genomics). Day 3 samples were processed using two wells of a Chromium Next GEM Chip G, and day 15 samples were processed using one well of a Chromium Next GEM Chip K.

Multimodal scRNA-seq was performed using the Chromium Next GEM Single Cell Reagent Kits (10× Genomics) according to the manufacturer’s instructions and modifications as previously published (32). The Chromium Next GEM Single Cell V(D)J reagent kits (v1.1) for Chip G and the Chromium Next GEM Single Cell 5′ Reagent Kits v2 for Chip K (10× Genomics) were used to prepare mRNA and surface protein libraries. cDNA amplification and target enrichment were quantified using a Qubit 3 Fluorometer (Invitrogen) and assessed for quality using a 4200 TapeStation System (Agilent). Libraries were constructed and sequenced on the NextSeq 2000 system (Illumina) to a target depth of 20,000 paired reads per cell for the gene expression and 5000 reads/cell for the surface protein libraries. The Cell Ranger ‘multi’ pipeline (v7.1.0; 10× Genomics) was used to conduct the alignment and feature expression quantification of the single-cell sequencing data. mRNA reads were aligned to the GRCh38 human reference genome. Hashtag oligo (HTO) and Ab-derived tag reads were aligned to a feature reference containing the appropriate barcode sequences. The day 3 Cell Ranger output was aggregated using the command-line tool cellranger aggr.

The single-cell gene expression and surface protein data were assessed for quality using the R packages Seurat (v5.0.1) (33) and scDblFinder (v1.14.0) (34). Data from day 3 and day 15 samples were processed separately using the following steps. HTO data were normalized using centered log-ratio transformation. Samples were demultiplexed based on HTO enrichment using the *MULTIseqDemux* (35) function, removing between-sample doublets and negative or ambiguous cell barcodes. Cell barcodes classified as singlets were filtered based on gene expression criteria (>200 unique genes/cell and <10% mitochondrial counts). Within-sample doublets were removed using scDblFinder. mRNA count data were normalized using the *SCTransform* function. Cells were clustered and visualized using the *RunPCA*, *FindNeighbors*, *RunUMAP*, and *FindClusters* functions. Cells were annotated with SingleR (v2.2.0) using the HumanPrimaryCellAtlasData reference and raw RNA counts (36). SingleR pruned labels were visually inspected in comparison with cell-type marker gene expression. Cells that could not be annotated by SingleR were labeled “Unknown.” Some labels were consolidated, and lymphoid and myeloid cells were reclustered together and reannotated using Azimuth (v0.5.0) (37). Neutrophils were not detected. SingleR and Azimuth labels were manually merged for final cell annotation. Ab-derived tag count data were normalized and background corrected based on mouse IgG isotype controls. Azimuth labels were visually inspected in comparison with cell-type marker gene expression. Cell-cell communication networks were inferred using CellChat (v2.1.1) (S. Jin, M. V. Plikus, and Q. Nie, manuscript posted on bioRxiv, DOI: 10.1101/2023.11.05.565674). Interactions shown are significant, are weighted by communication strength, consider the population size of each cell type, and exclude extracellular matrix pathway interactions. Module scores were calculated for genes enriched in neutrophil (*n* = 66) and monocyte (*n* = 67) blood transcription modules using the Seurat function *AddModuleScore* with nbin = 18. The chosen neutrophil module genes are LIN7A, NCF4, EMR3, FCGR3A, S100P, FCGR3B, TNFRSF10C, C5AR1, MGC31957, CHI3L1, NLRP12, CYP4F3, MXD1, SEPX1, MGAM, DGAT2, RGL4, REPS2, VNN2, CXCR1, NFE2, KRT23, GPR109B, PYGL, FPR2, G0S2, KCNJ15, LRRC4, FPR1, CREB5, FCGR2A, CMTM2, MANSC1, CSF3R, FFAR2, RNF24, LRG1, ST6GALNAC2, ORM1, MME, CDA, PROK2, PFKFB4, VNN3, SLC22A4, BASP1, TREM1, GLT1D1, GPR97, PTAFR, STEAP4, ALPL, NPL, ARAP3, HSPA6, TYROBP, CFD, IMPA2, DENND3, BTNL8, FRAT2, MBOAT7, TSPAN2, BEST1, FLJ10357, and SLC40A1. The chosen monocyte module genes are TNFSF13, MYCL1, EPB41L3, DPYSL2, RTN1, SLC31A2, FES, LGALS3, HCK, APLP2, LGALS1, PTGS2, EMR1, AMICA1, DOCK5, CD4, LY96, ARHGEF10L, TNFSF13B, LTBR, PGD, TNFRSF1B, LRRK2, DPYD, MGAM, PTX3, LYZ, IL1R2, DOK3, CARD9, EVI5, GPR109B, DUSP6, MYO1F, FGD4, HHEX, HAL, ST3GAL6, DYSF, RNASE6, SLC24A4, VNN1, NAIP, RHOU, CD68, CXCR2, NACC2, SMARCD3, PADI4, TMEM176B, SAMHD1, CTSS, EMILIN2, ACPP, F5, STEAP4, C19orf59, ACSL1, PAK1, C1orf162, MOSC1, TLR1, PID1, BCL6, HLA-DMB, MPP1, and AGPAT9.

### Mass cytometry by time of flight

Cells were plated in a 96-well U-bottom plate at ∼170,000 to 1.6 million cells/well and washed twice with PBS. Cells were then stained with Cell-ID Cisplatin (Fluidigm) and incubated for 5 min at room temperature. Cells were washed twice with FACS buffer and stained with MR1 5-OP-RU allophycocyanin tetramer (NIH Tetramer Core Facility) for 1 h at room temperature, followed by three washes with Maxpar Cell Staining Buffer (Fluidigm). Next, cells were stained with a mixture of the following surface Abs for 30 min at room temperature: anti-CD68 106Cd (clone KP1; BioLegend), anti-CD8β 113Cd (clone 2ST8.5H7; Novus Biologicals), anti-CD66abce 141Pr (clone Tet2; Thermo Fisher Scientific), anti-CD69 145Nd (clone FN50; BioLegend), anti-CD127 149Sm (clone A019D5; Fluidigm), polyclonal anti-IgD 153Eu (SouthernBiotech), anti-CD161 160Gd (clone HP-3G10; BioLegend), pan anti-TCR7γδ 171Yb (clone B1; BioLegend), anti-CD45RA 174Yb (clone 5H9; BD Biosciences), anti-CD28 175Lu (clone CD28.2; BioLegend), anti-allophycocyanin 163Dy (Fluidigm), anti-CD11b 144Nd (clone ICRF44; Fluidigm), anti-CD11c 146Nd (clone 3.9; Fluidigm), anti-CD20 147Sm (clone 2H7; Fluidigm), anti-CD14 151Eu (clone M5E2; Fluidigm), anti-CD163 154Sm (clone GHI/61; Fluidigm), anti-CXCR3 156Gd (clone G025H7; Fluidigm), anti-CCR7 159Tb (clone G043H7; Fluidigm), anti-NKG2A 169Tm (clone Z199; Fluidigm), anti-CD8α 111Cd (clone RPA-T8; BioLegend), anti-CD24 112Cd (clone SN3; Thermo Fisher Scientific), anti-CD4 114Cd (clone L200; BD Biosciences), anti-CD45 116Cd (clone D058-1283; BD Biosciences), anti-CD123 161Dy (clone 6H6; BioLegend), anti-CD206 172Yb (clone 19.2; BD Biosciences), anti-CD16 209Bi (3G8; Fluidigm), and anti–HLA-DR 176Yb (clone LN3; BioLegend). After three washes with Maxpar Cell Staining Buffer (Fluidigm), cells were resuspended in Fix 1 buffer and incubated for 20 min at room temperature. Cells were washed twice with Maxpar Perm-S Buffer (Fluidigm) and stained with a mixture of the following intracellular Abs for 30 min at room temperature: anti-CD154 162Dy (clone 24–31; BioLegend), anti-Perforin 164Dy (clone Pf-80/164; Mabtech), anti-Granzyme K 165Ho (clone GM26E7; BioLegend), anti–IL-10 166Er (clone JES3-9D7; Fluidigm), anti–IL-2 158Gd (clone MQ1-17H12; Fluidigm), anti–Granzyme B 173Yb (clone GB11; Fluidigm), anti-CXCL10 150Nd (clone J034D6; BioLegend), anti-CCL4 142Nd (clone 24006; R&D Systems), anti–IL-1β 155Gd (clone JK1B-1; BioLegend), anti–IL-6 167Er (clone MQ2-6A3; BD Biosciences), anti–IL-17A 148Nd (clone BL168; Fluidigm), anti–IFN-γ 168Er (clone B27; Fluidigm), anti-TNF 152Sm (clone Mab11; Fluidigm), and anti-CD3 170Er (clone SP34-2; Fluidigm). After three washes with Maxpar Perm-S Buffer (Fluidigm), cells were fixed with 2% paraformaldehyde. Cells were then washed with Maxpar Perm-S Buffer (Fluidigm) and resuspended in a mixture of Cell-ID Intercalator-Ir (Fluidigm) and Maxpar Fix and Perm Buffer (Fluidigm). Samples were stored at 4°C until acquisition. Fixed samples were acquired on a Helios mass cytometer (Fluidigm).

Cytometry by time-of-flight (CyTOF) data were gated up to CD45^+^ cells using FlowJo (v10) (BD Life Sciences). Live CD45^+^ cells were then clustered and analyzed using the R packages FlowSOM (v2.8.0) (38) and CATALYST (v1.24.0) (39) following the R-based pipeline described by Nowicka et al. (40). Marker intensities were arcsinh transformed with a scaling factor of 5 to make the distributions more symmetric. Nonredundancy scores were calculated to determine the ability of each marker to explain the variance observed at each time point. The following surface markers did not stain very well and were excluded from clustering because they had the lowest nonredundancy scores: NKG2A, CD24, CD4, CD8α, CD69, CD206, and CD8β. The data were then clustered on phenotypic marker intensities and manually annotated. Wilcoxon rank-sum tests with unadjusted *p* values were used to compare frequencies of cell types between groups.

### Data availability

Code that was generated to produce analysis and figures for this article is available on GitHub (https://github.com/seshadrilab/bcg-dermal-immunity). The raw sequencing data presented in this article have been submitted to the NCBI Sequence Read Archive under accession numbers PRJNA1148666 and PRJNA1150210. Cytometry time-of-flight (CyTOF), flow cytometry intracellular cytokine staining (ICS), binding Ab multiplex assay (BAMA), and processed sequencing data are available on Zenodo (https://doi.org/10.5281/zenodo.13737809).

## Results

### Participant demographics and AEs

**FIGURE 1.**
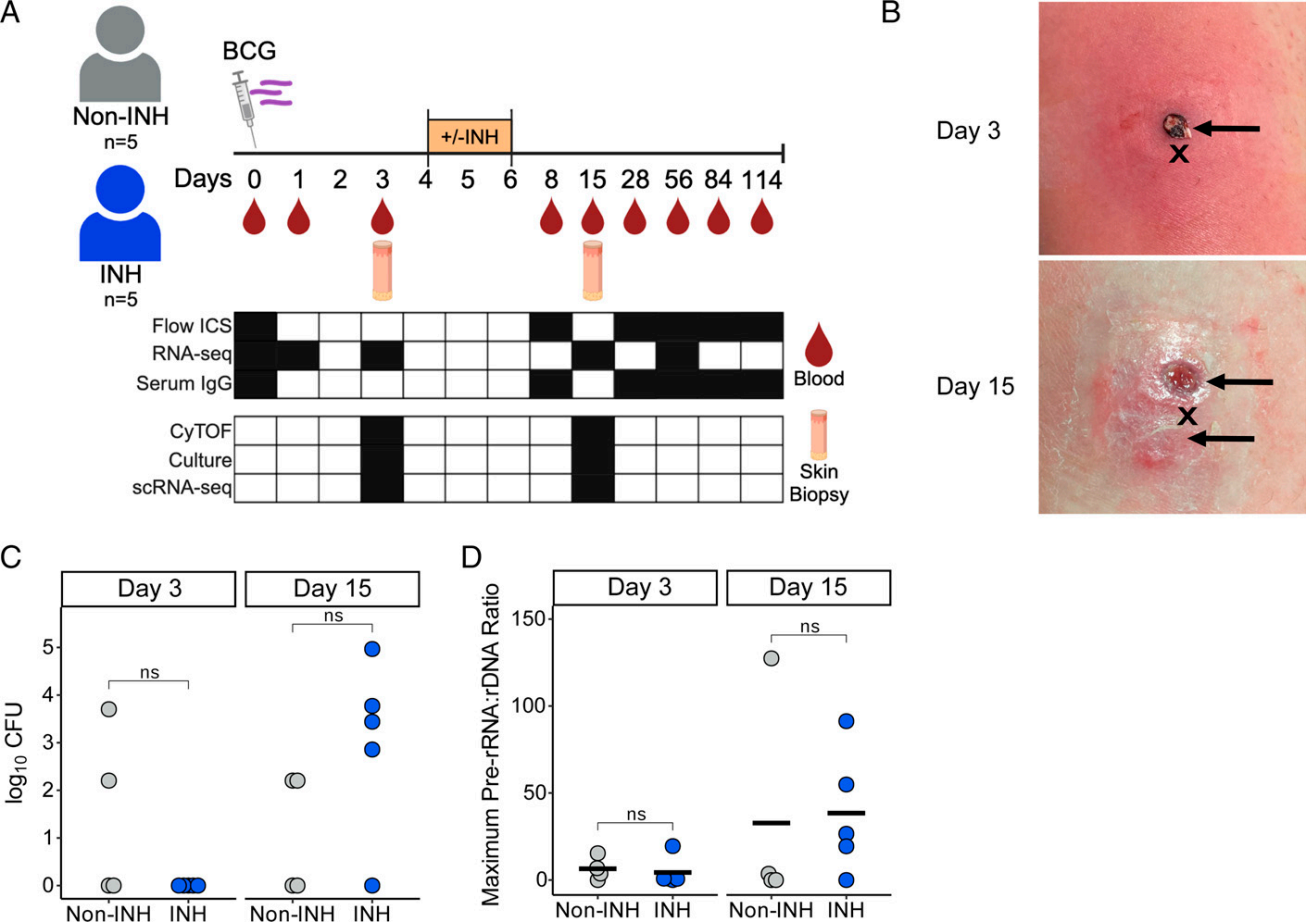
Study schema and primary microbiology outcomes. (**A**) Study schema. Ten participants received intradermal BCG, and half were randomized to receive INH therapy. Blood samples were collected at multiple time points, and skin punch biopsies were collected at days 3 and 15 post-BCG injection. (**B**) Photos of the injection site at days 3 and 15 from a representative participant. Biopsy sites (arrows) are located 2–4 mm above and below the injection site (“X”). Day 15 photo was taken prior to biopsy. (**C**) CFUs on 7H10 solid media. (**D**) Maximum pre-rRNA/rDNA ratio (MVT), which indicates the presence of viable bacteria. (C and D) Results for INH-treated (*n =* 5) and untreated (*n =* 4) participants. Significance comparing treatment arms was determined by two-sample, two-sided, unpaired Wilcoxon test.

A total of 10 participants were enrolled between November 3, 2022, and December 1, 2022 (Table I). Participants were randomly assigned to receive INH versus no treatment (Fig. 1A). All participants received BCG vaccinations, and all participants underwent day 3 skin biopsies. Nine of 10 participants underwent day 15 skin biopsies, with one of those participants being excluded because of being unable to present during the scheduled visit day. The median age at enrollment was 36 y (range 20–43 y). Notably, the median age was significantly different between the two groups (*p* = 0.035), with participants receiving INH having a median age of 28 y (range 20–36 y) and participants not receiving INH having a median age of 40 y (range 29–43 y). Six (60%) participants were male, three (30%) were female, and one (10%) was intersex. Eight (80%) participants were white, one (10%) was Black, and one (10%) was mixed race.

**Table I.** Demographic characteristics of study participants

	INH (*n* = 5)	Non-INH (*n* = 5)	Total (*N* = 10)
Sex			
Male	3 (60%)	3 (60%)	6 (60%)
Female	2 (40%)	1 (20%)	3 (30%)
Intersex	0	1 (20%)	1 (10%)
Ethnicity			
Hispanic or Latino/a	1 (20%)	0	1 (10%)
Not Hispanic or Latino/a	4 (80%)	5 (100%)	9 (90%)
Race			
White	4 (80%)	4 (80%)	8 (80%)
Black	0	1 (20%)	1 (10%)
Mixed	1 (20%)	0	1 (10%)
Age, y			
18–20	1 (20%)	0	1 (10%)
21–30	2 (40%)	1 (20%)	3 (30%)
31–40	2 (40%)	0	2 (20%)
41–45	0	4 (80%)	4 (40%)
Median	28	40	36
Range	20–36	29–43	20–43

Numbers of donors, sex, race/ethnicity, and age are indicated. Randomization to INH or non-INH treatment arm was performed using random number generators, and once one arm was filled, participants were assigned to fill the remaining arm. The median age at enrollment was 36 y (range 20–43 y), with a significant (*p* = 0.035) difference in median age between the INH (median = 28) and non-INH (median = 40) arms.

All participants had erythema and induration at the inoculation site post-BCG vaccination. Two (20%) participants experienced grade 3 erythema (size > 10 cm). Only one participant reported a severe AE (SAE) of back pain, which was determined to be unrelated to the study. One participant experienced grade 1 lymphadenopathy. All other study-related AEs consisted of mild fatigue, headache, and adhesive allergies. No SAEs related to the study were reported (Table II).

**Table II.** Adverse events

	Any Grade	Grade ≥ 3	SAEs
Population	10	10	10
Any AEs	10 (100%)	3 (30%)	1 (10%)
Pyrexia	0	0	0
Chills	0	0	0
Fatigue	1 (10%)	0	0
Pain	2 (20%)	0	0
Tenderness	9 (90%)	0	0
Erythema	10 (100%)	2 (20%)	0
Induration	10 (100%)	0	0
Pruritus	8 (80%)	0	0
Lymphadenopathy	1 (10%)	0	0
Rash (adhesive related)	7 (70%)	0	0
Myalgia	0	0	0
Arthralgia	0	0	0
Back pain	1 (10%)	1 (10%)	1 (10%)
Other joint pain	1 (10%)	0	0
Other			
COVID-19 infection	2 (20%)	0	0
Upper respiratory tract infection	2 (20%)	0	0
Headache	3 (30%)	0	0

AEs experienced by participants are indicated. Only one participant reported an SAE of back pain, which was determined to be unrelated to the study.

### Primary microbiologic outcomes

Biopsies obtained at days 3 and 15 were split for primary microbiologic analysis and immune profiling (Fig. 1A, 1B). Culture and molecular viability data were available for 10 participants on day 3 and 9 participants on day 15 (INH = 5, non-INH = 4). Initial solid culture of residual reconstituted vaccine yielded 5.01 × 10^4^ CFUs on polymyxin B, amphotericin B, nalidixic acid, trimethoprim, and azlocillin–containing media, which represented a 40-fold reduction compared with 2 × 10^6^ CFUs indicated on the vial. No bacteria were cultured from the pretreatment INH group at day 3, and no significant difference was seen between culture results from day 15 (posttreatment) in the INH versus non-INH groups (Fig. 1C). We also assessed bacterial viability using molecular assays. Pre-rRNA/rDNA ratio was positively correlated with number of CFUs with a Kendall rank correlation coefficient (τ) of 0.47 (*p* = 0.02) (Supplemental Fig. 1A). However, the pre-rRNA/rDNA ratio did not show a difference between treatment groups at day 15 (Fig. 1D). These results show that 3 d of INH treatment immediately after receiving intradermal BCG did not have an effect on bacterial growth or viability on day 15. Due to the lack of effect of INH on bacterial burden, results of INH and non-INH treatment groups were pooled for immunologic analyses.

### Peripheral blood immune response seen as early as 8 d post-BCG

**FIGURE 2.**
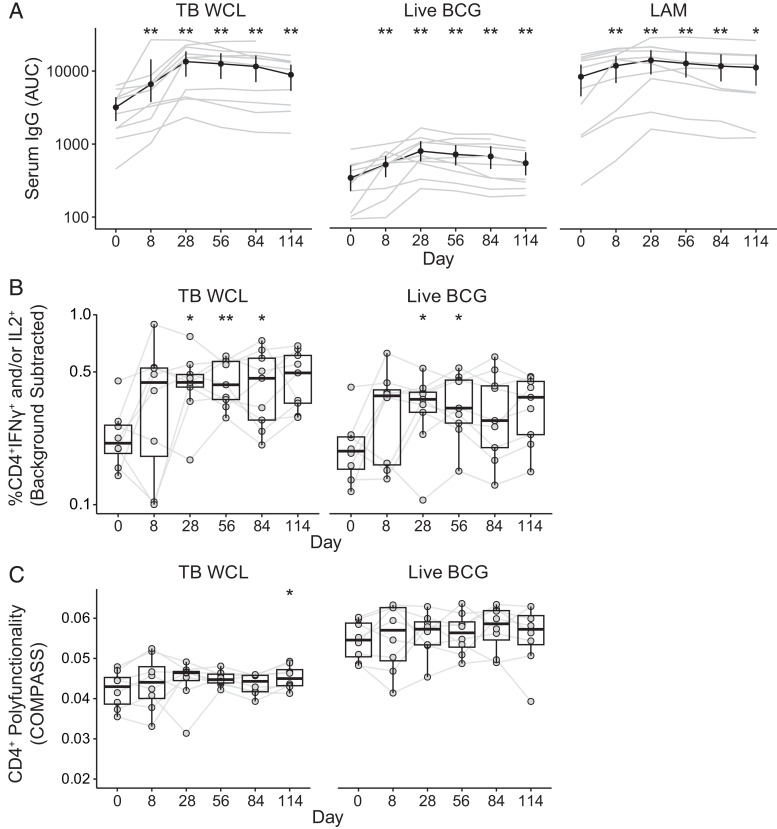
Peripheral immune responses seen as early as 8 d post-BCG. Results from serum binding Ab multiplex assay (BAMA), flow cytometry ICS, and flow cytometry COMPASS analysis. (**A**) Area under the curve (AUC) of serum IgG binding levels in response to *M. tuberculosis* WCL (TB WCL), live BCG Tice, or *M. tuberculosis* LAM as measured by BAMA (*n =* 10 for days 0, 8, 28, 56, and 84; *n =* 9 for day 114). Black lines indicate median values across participants. (**B**) Proportion of CD4^+^ T cells expressing IFN-γ and/or IL-2 as measured by ICS after stimulation with TB WCL or live BCG Tice. (**C**) COMPASS PFSs in CD4^+^ T cells after stimulation with TB WCL or live BCG. PFS reflects the proportion of cells expressing a range of cytokines, weighted by the number of cytokines expressed per cell. (B and C) Boxplots represent the median and interquartile range. Gray lines indicate the same individual across time points. *n =* 8, days 0 and 8; *n =* 9, days 28, 56, 84, and 114. Statistical significance comparing results to day 0 was calculated using paired Wilcoxon signed rank tests. *Unadjusted *p* < 0.05, **unadjusted *p* < 0.01.

We next sought to examine the breadth of peripheral immune responses to BCG. We used a bead-based multiplex assay to quantify binding IgG Ab responses to BCG Tice, LAM, and *M. tuberculosis* WCL (41). We observed an increase in responses to all three Ags as early as 8 d after BCG inoculation, with peak responses at approximately day 28 and no differences when stratified by treatment group (Fig. 2A and data not shown). We then examined the peripheral blood T cell response using a qualified 28-parameter ICS flow cytometry panel. PBMCs were stimulated with BCG Tice, *M. tuberculosis* WCL, or PBS as a negative control. Similar to IgG responses, we observed an increase in the frequency of Ag-specific CD4^+^ T cells producing IFN-γ and/or IL-2 8 d post-BCG (Fig. 2B). The magnitude of CD8^+^ responses was 10-fold lower and did not change appreciably after BCG (Supplemental Fig. 1B). We employed COMPASS to compute PFSs for CD4 (Fig. 2C) and CD8 (Supplemental Fig. 1C) T cells in response to WCL or BCG Tice (26). We observed an increase in the CD4 T cell PFS that mirrored the magnitude of cells expressing IFN-γ and/or IL-2 (Fig. 2C). Polyfunctional CD4 T cells commonly expressed GM, CD153, IL-2, IFN-γ, TNF, and/or CD154 at levels greater than the negative control and did not differ by treatment group (data not shown).

### Genes related to monocytes and neutrophils are downregulated in whole blood at day 56 post-BCG

**FIGURE 3.**
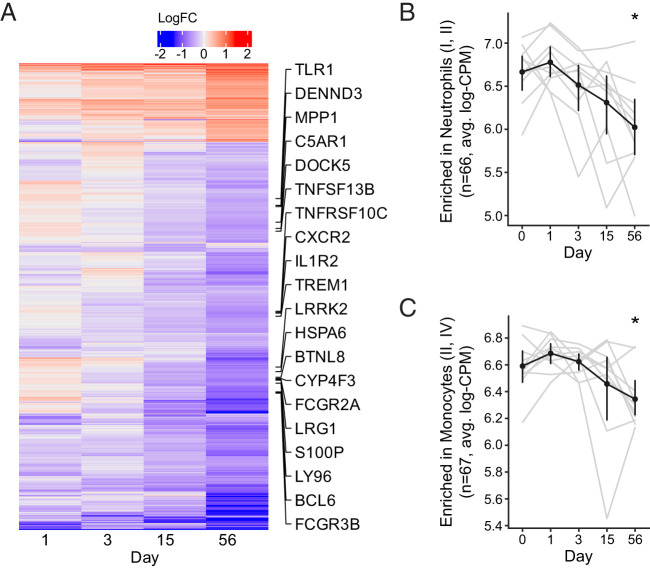
Genes related to monocytes and neutrophils are downregulated in whole blood at day 56 post-BCG. Bulk RNA-seq of whole blood was performed at multiple time points (*n =* 10). (**A**) Heatmap showing log_2_FC gene expression for all 1497 differentially expressed genes (*p* < 0.05, FDR < 0.05, and absolute log_2_FC > 0.5), averaged across participants. A subset of monocyte and neutrophil BTM genes are labeled LogFC = log_2_FC. (**B** and **C**) Average normalized expression of all genes in the (B) neutrophil (I, II) and (C) monocyte (II, IV) BTMs. Black lines indicate median values across participants. Statistical significance comparing results with day 0 was calculated using paired Wilcoxon signed rank tests. *Unadjusted *p* < 0.05.

Next, we examined the blood transcriptome response to intradermal BCG challenge. Whole blood collected before and at serial time points after BCG was subjected to bulk RNA-seq. We used linear mixed modeling to compare each time point post-BCG with baseline using participants as the grouping variable. We observed upregulated and downregulated DEGs at all post-BCG time points; however, we observed the greatest number at day 56 with 1198 genes downregulated (Fig. 3A). We speculated that the response could be linked to an intercurrent illness unrelated to vaccination. However, review of the clinical data confirmed that 8 of the 10 participants did not report any illnesses near the time of this study visit. One participant reported COVID-19 on day 45 and another reported a sinus infection on day 57. Further, when comparing visit pairs, no DEGs were found between days 15 and 56, suggesting that significant gene changes by day 56 may already have been present by day 15 (data not shown).

We used gene set enrichment testing to determine whether the DEGs at each time point were associated with previously established vaccine-related BTMs. Gene set enrichment was observed only for downregulated genes at days 15 (5 gene sets) and 56 (24 gene sets). The gene sets that were enriched at day 15 had functions related to erythrocyte differentiation (M173, FDR-q = 0.032), heme biosynthesis (M171, M222, FDR-q = 0.032), and transcriptional regulation (M49, FDR-q = 0.032). The gene sets enriched at day 56 included functions related to neutrophils, monocytes, dendritic cells, and immune activation. For example, 60 genes were enriched in three respective neutrophil modules (M37.1, M132, M163, all FDR-q < 0.0001). Gene module expression was significantly lower at day 56 compared with day 0 for the modules “Enriched in Neutrophils (I, II)” (Fig. 3B, *p* = 0.01), “Enriched in Monocytes (II, IV)” (Fig. 3C, *p* = 0.04), and “TLR and inflammatory signaling (M16)” (not shown, *p* = 0.01). The decrease in expression of these genes may either indicate a decrease in their activation or a migration of these cell types out of peripheral blood and into the tissues.

To better understand the kinetics of the gene expression responses, we used the significantly enriched gene sets to compute normalized expression scores for each participant over time (Fig. 3B, 3C). With each gene set we were able to see how known groups of coregulated genes responded to BCG. For example, the monocyte gene sets showed a small increase at day 1, characteristic of an innate response, that was not otherwise detectable among individual genes. The monocyte gene sets then decreased markedly by day 56 (Fig. 3C).

### Immune cell frequencies at injection site change over time

**FIGURE 4.**
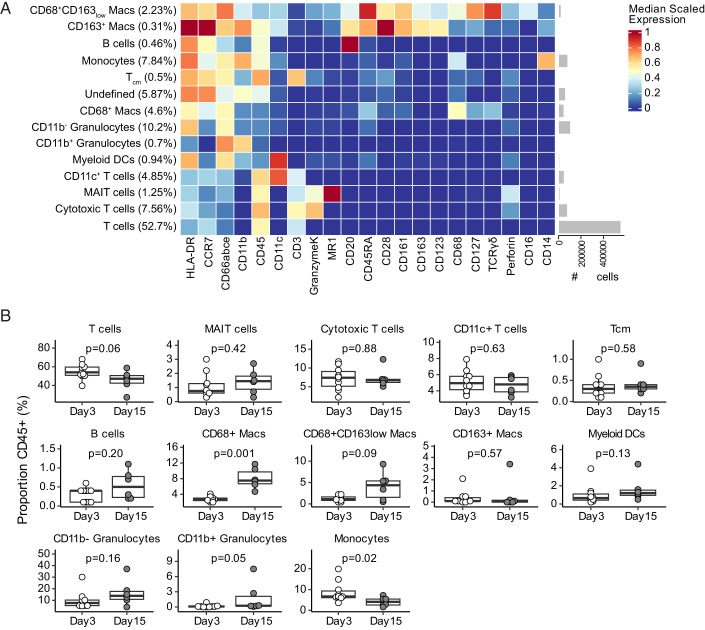
Immune cell frequencies at injection site change over time. Skin biopsies were subject to assays including CyTOF, using an immune-focused panel. Immune cell types present at the injection site and changes in their frequencies between days 3 and 15 were identified by CyTOF. (**A**) CD45^+^ cell subsets were identified using the FlowSOM clustering algorithm and manually annotated (*n =* 18). “Undefined” represents CD45^+^ cells that could not be confidently annotated. (**B**) Cell subset frequencies as a fraction of total CD45^+^ cells were calculated at each time point (day 3, *n =* 10; day 15, *n =* 6). Samples with low cell counts (day 15 samples for BCG05 and BCG08) were excluded. Comparisons were made using unpaired Wilcoxon rank-sum test with unadjusted *p* values displayed.

We examined the in situ response to BCG with a focus on changes in immune cells from days 3 to 15. Skin biopsy specimens dedicated for immune profiling were subjected to digestion and single-cell isolation. Biopsies were available from 10 participants on day 3 and 9 participants on day 15 (INH = 5, non-INH = 4). Cells were analyzed using a 46-parameter CyTOF panel, which was largely focused on immune cell subsets. Total CD45^+^ cells available for analysis ranged from 135 to 167,165 (Supplemental Fig. 3A), and initial quality-control filtering revealed that seven markers were not informative likely because of cleavage during the tissue digestion step (Supplemental Fig. 3B). We analyzed the remaining 21 markers using FlowSOM and identified 14 distinct cell subsets (Fig. 4A) (38). All CD45^+^ cells were identified by manual gating in FlowJo (Supplemental Fig. 3C). All leukocyte populations were first identified as CD45^+^. Macrophages were identified by CD68^+^ and/or CD163^+^, and monocytes by CD14^+^ and lack of expression of macrophage markers. CD3 was used to identify T cells and CD20 to identify B cells. Neutrophils were identified by the expression of CD66abce with or without CD11b expression, and myeloid dendritic cells were identified as HLA-DR^+^CD11c^+^CD123^−^. We were able to further classify T cells into MAIT cells using MR1-5-O-PRU tetramer^+^, cytotoxic T cells using GranzymeK^+^, and CD11c^+^ T cells using CD11c and CD3. We could not identify CD4^+^, CD8^+^ T cells, and NK cells (NKG2A^+^CD3^+^) because CD4, CD8, and NKG2A were excluded from analysis because of low nonredundancy scores. Undefined cells are CD45^+^ cells that could not be assigned as one of the classified subsets (5.87% of CD45^+^). The most frequently observed cells were T cells (52.7%), followed by CD11b^−^ granulocytes (10.2%) and monocytes (7.84%). Several other lymphoid and myeloid populations were observed at much lower frequencies as well. We next examined whether the frequency of any FlowSOM subsets changed between days 3 and 15 (Fig. 4B). The frequencies of T cells, monocytes, and undefined cells decreased between time points (*p* = 0.01, *p* = 0.02, and *p* = 0.01, respectively), whereas frequencies of CD68^+^ macrophages, CD68^+^CD163^low^ macrophages, and CD11b^−^ granulocytes increased (*p* = 0.01, *p* = 0.01, and *p* = 0.08, respectively). Together, these data reveal dynamic leukocyte population changes in the skin after BCG challenge, most notably among myeloid cells.

### scRNA-seq reveals changing myeloid expression and cross-talk at injection site

**FIGURE 5.**
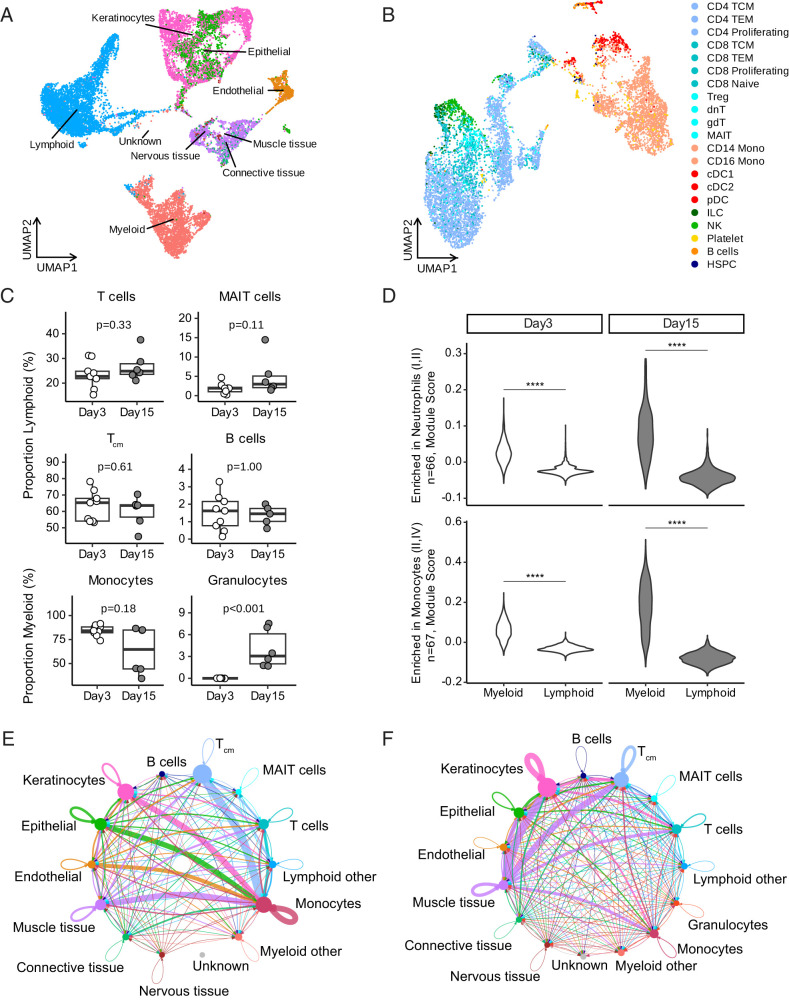
scRNA-seq reveals changing myeloid expression and cross-talk at injection site. (**A**) All cells that passed quality filtering were automatically annotated on a cell-cell basis using the SingleR package with the “HumanPrimaryCellAtlasData” reference. “Unknown” represents unable to annotate (day 3 = 12,178 cells, day 15 = 4731 cells). (**B**) Lymphoid and myeloid cells were subclustered and automatically annotated using the Azimuth package with the “pbmcref” reference (day 3 = 6662 cells, day 15 = 2090 cells). (A and B) For visualization purposes, all cells were integrated using Harmony and are shown in UMAP space (*n =* 15). Notably, no day 3 cells could be annotated as granulocytes and so these do not appear in the integrated data shown here. (**C**) Select immune cell proportions as a fraction of total parent cell type (lymphoid or myeloid) at days 3 (*n =* 9) and 15 (*n =* 6). Statistical testing was performed with unpaired Wilcoxon rank-sum test, and unadjusted *p* values are displayed. (**D**) Violin plots of module scores, a measure of relative expression of a gene set, for genes enriched in neutrophil (top) and monocyte (bottom) blood transcription modules from Fig. 3B and 3C at days 3 (*n =* 9) and 15 (*n =* 6). Statistical significance was calculated using two-sided Wilcoxon rank-sum tests, and results were adjusted for multiple comparisons with the Bonferroni method. ****Adjusted *p* < 0.0001. (**E** and **F**) Cell-cell interactions were identified by CellChat at (E) day 3 (*n =* 9) and (F) day 15 (*n =* 6). Significant interactions were identified using a permutation test and *p* < 0.05. All interactions shown are weighted by communication strength and scaled to maximum strength for that day. Extracellular matrix pathway interactions are not shown. Notably, no day 3 cells could be annotated as granulocytes.

In addition to mass cytometry, we performed multimodal scRNA-seq on the skin biopsy specimens. We generated scRNA-seq data for all but five biopsies (one from the day 3 INH group and four from the day 15 non-INH group). The gene expression count matrices from Cell Ranger filtered feature output were used to perform cell clustering and annotation. A total of 12,178 and 4731 cells passed quality control at days 3 and 15, respectively (Supplemental Fig. 4A, 4B). All cells that passed quality control from each time point were clustered. We did not observe cells clustering by time point, participant, or treatment arm when visualized in uniform manifold aproximation and projection (UMAP) space (Supplemental Fig. 4C–E). All cells were annotated automatically using SingleR. All donors contributed to each cell type. We detected lymphoid, myeloid, keratinocyte, epithelial, muscle, endothelial, nervous, and connective tissue cells at both time points (Fig. 5A). Cells that could not be annotated were labeled “Unknown.” Lymphoid cells made up the largest population at both time points. We next subclustered lymphoid and myeloid cells together and annotated automatically using Azimuth. There were 6662 and 2090 immune cells at days 3 and 15, respectively. We detected B and NK cells, platelets, and multiple populations of T cells, monocytes, and dendritic cells, among others (Fig. 5B). For both Azimuth and SingleR, annotations were checked against expression of key marker genes (Supplemental Fig. 4F, 4G).

We measured changes in immune cell–type population frequencies between days 3 and 15 as a fraction of total lymphoid or myeloid cell count (Fig. 5C). We observed a significant increase in granulocyte frequency (*p* < 0.001) and a trend toward a decrease in monocyte frequency (*p* = 0.18). Both of these observations were consistent with our CyTOF findings (Fig. 4B). We also measured the expression of genes from the “Enriched in Neutrophils (I, II)” and “Enriched in Monocytes (II, IV)” gene modules identified in the blood transcriptome (Fig. 3B, 3C). Relative expression of these genes was significantly higher in myeloid cells compared with lymphoid cells at both days (Fig. 5D).

Finally, we examined cellular cross-talk using CellChat (Fig. 5E, 5F). We excluded extracellular matrix signaling and visualized significant interactions weighted by communication strength, which was inferred while accounting for the number of cells in each annotation type. At day 3, signaling was predominantly directed from muscle, endothelial, epithelial, keratinocyte, and T central memory (T_CM_) cells toward myeloid cells, which were also communicating among themselves (Fig. 5E). At day 15, there was more cross-talk occurring in general and between all cell types, with an emphasis on communication from muscle cells to keratinocytes and T_CM_ cells, and keratinocytes among themselves. We were intrigued by the shift away from monocytes and investigated the top pathways involving monocytes at each day. The migration inhibitory factor (MIF)–(CD74+CD44), MIF–(CD74+CXCR4), and HLA-E–KLRK1 ligand–receptor interaction pairs dominate monocyte signaling at both days (data not shown), with MIF–(CD74+CD44) being involved in communication directed from T_CM_ cells to monocytes. HLA-E–KLRK1 interaction was observed strongly at both days with some variation in communication strength between time points. At day 3, we observed strong monocyte-to-monocyte interaction via MIF–(CD74+CD44), which was not observed at day 15. At day 15, we observed strong monocyte-to-T_CM_ interaction via MIF-(CD74+CXCR4) and keratinocyte-to-monocyte interaction via MIF–(CD74+CD44), neither of which is seen at day 3. Taken together, these data highlight the contribution of nonimmune cells in the dermal immune response to BCG.

## Discussion

Prior controlled human infection studies using intradermal BCG have focused on conventional microbiologic assessments, such as quantification of bacterial burden using CFUs or PCR (12, 42). Our study advances work in this field in three ways. First, we employed a new assay to measure microbial viability in skin lesions. The results were largely concordant and provided complementary information to CFU measurements, although conclusions are limited because of small sample size and lack of viable mycobacteria in several samples. Second, we measured peripheral blood immune responses at both early (days 1–15) and late (days 56–114) time points, which allowed us to examine early innate responses and subsequent adaptive immunity. Finally, we performed serial biopsies and used CyTOF and multimodal scRNA-seq to comprehensively examine immune responses at the site of inoculation. Our results reveal a diversity of immune and nonimmune cell types engaged in the dermal immune response to cutaneous mycobacterial infection.

Our findings regarding the peripheral blood immune response to intradermal BCG confirm and extend prior work in the field. A study comparing BCG-vaccinated or BCG-naive adults in the United Kingdom examined correlations between gene expression profiles in PBMCs and BCG in skin biopsies obtained 2 wk after challenge (43). They found that IFN-γ and IL-17 pathways were strongly induced in participants who were previously vaccinated with BCG, and this correlated with inhibition of mycobacterial growth. A South African study led by members of our team revealed peak T cell and binding Ab responses at 10 wk postvaccination with 2–8 × 10^5^ CFUs of BCG (44). Surprisingly, we observed adaptive immune responses as early as 8 d postinoculation in a BCG-naive population. The 8 d time point was not tested in the South African study. Notably, responses to Ag-specific IgGs are noted in all participants at baseline, likely because of exposure to environmental mycobacteria. A more recent study employed multiple noninvasive techniques to quantify bacterial burden after dose-ranging intradermal BCG challenge, and examined transcriptional signatures from whole blood (14). They reported modest changes in gene expression at the 2 × 10^6^ dose, which largely normalized by day 43. By contrast, we report few changes on days 1, 3, and 15 compared with baseline, but nearly 1200 DEGs by day 56. As described earlier, we do not have any evidence that this was driven by concurrent infections, and a gene module associated with these genes did change over time. Without a control group that did not receive BCG, it is difficult to determine whether these changes were due to the vaccine. However, prior work has demonstrated that viable BCG can be recovered from the skin up to 56 d postinoculation (45).

Despite a significant body of work focused on studying systemic immune responses to BCG or *M. tuberculosis*, very little is known about the local immune response in the setting of cutaneous mycobacterial infections. A study of 15 leprosy skin lesions used dual RNA-seq to study both bacterial and host transcriptomes, including an assessment of molecular viability analogous to what we have done in this study (46). They found that bacterial burden was positively correlated with type I IFN gene signature, whereas bacterial viability was associated with the expression of bacterial heat shock proteins and activation of the host Ab pathway. Another study using single-cell and spatial sequencing approaches described the cellular architecture of leprosy granulomas and identified antimicrobial response genes in reversal reactions that were regulated by IFN-γ and IL-1β (47). Our work extends these data by providing a detailed assessment of immune responses to BCG in skin over time. Of interest, there were several nonimmune cell types, such as endothelial, epithelial, and muscle cells, that appeared to be involved in recruiting and communicating with the immune response to BCG, which merits further investigation. Airway epithelial cells have been shown to participate in an immune network with *M. tuberculosis–*infected alveolar macrophages through the expression of antimycobacterial peptides, defensins, and S100-family proteins (48). In a zebrafish model, granuloma macrophages appeared to be reprogrammed by epithelial cells (49). Epithelial cells can also become productively infected and activate local CD8 T cells to secrete IFN-γ (50). Vascular endothelial cells respond to signals from *M. tuberculosis*–infected myeloid cells to promote angiogenesis, which is important for granuloma formation and bacterial dissemination (51). The role of muscle cells in mycobacterial pathogenesis is undefined. However, skeletal muscle can modulate the function of both myeloid and T cells through production of IL-6 and glutamine (52). Conversely, skeletal muscle cells express receptors for IL-1, IL-6, and IFN-γ, as well as chemokine receptors such as CCR2, CCR4, and CCR10 (53). These data can inform future investigation into the immunology and host responses to BCG, *M. tuberculosis*, and other cutaneous mycobacteria and advance research into treatments and host immune responses.

In this study, we did not see a reduction in BCG bacterial burden in skin in participants who received INH compared with those who did not. There are several potential explanations for this outcome. Because INH predominately acts by inhibiting synthesis of cell-wall components, the antibiotic is most potent on rapidly dividing mycobacteria (54). Expression of genes associated with mycobacterial growth inhibits expression of the activator *katG* transcription, leading to growth phase–dependent tolerance to INH (55, 56). BCG Tice is provided in a lyophilized form, which we reconstituted and immediately inoculated into the forearms of participants. As such, it is unlikely that BCG was in a physiologic state that would render it susceptible to INH. In addition, INH has demonstrated poor penetration into granulomas and tissue from blood relative to other anti-TB medications and may require a longer treatment time to achieve results than was given in this study (57). Finally, the group not receiving INH had a much older median age relative to the INH group (40 versus 28 y, respectively). Thus, baseline differences in the immune response to mycobacteria may have confounded comparisons between the treatment groups.

We were surprised by the limited recovery of viable BCG from the inoculation site. Although the target injection was 2 × 10^6^ CFUs, cultures taken from the residual in the vial showed ∼40-fold fewer CFUs than anticipated. There are reports of varying bacterial viability between licensed formulations of BCG (58). In addition, it has been long known that extended storage time and higher temperatures reduce bacterial viability (59). In our study, all participants were inoculated from a single vial that was stored according to the manufacturer’s recommendations and used before the target expiry date. It is also possible that biopsies were not performed at the site of maximum bacterial burden. In general, biopsies were collected when a large amount of erythema was present, and determining the inoculation site was difficult. This was reflected by the significant variability in CFUs at day 3 before treatment with INH. We also cannot rule out the possibility that BCG was rapidly cleared from the injection site. A mouse model of contained *M. tuberculosis* infection revealed that when 1 × 10^5^ CFUs are inoculated into the ear, viable bacteria could be detected in the draining lymph node within 5 d (60). Lastly, participants were all noted to have some existing baseline immune responses to mycobacteria, possibly related to prior exposure to environmental mycobacteria. Prior immunity could also have reduced the bacterial burden in the skin at the time of sampling.

Because we do not currently have a safe way to conduct challenge trials using *M. tuberculosis*, BCG remains a safe alternative challenge agent at this time. With further research into the skin-specific immunology when intradermal BCG is used, the scope of the challenge model could be expanded from vaccines to therapeutics, including host-directed therapies. For example, there has been recent interest in statins improving drug distribution and reversing mycobacterial-induced inhibition of phagolysosome-based autophagy in macrophages (61–63). A small challenge trial could be used to test multiple dose and statin varieties within a single cohort. Continued research into the local and systemic immune responses of BCG has substantial value in establishing a challenge method to be used in future TB vaccine and therapeutics trials and shorten the pathway to phase III clinical trials of promising interventions.

## Supplementary Material

Supplemental Material (PDF)
