# Supplementary material for: Probing Dermal Immunity to Mycobacteria through a Controlled Human Infection Model
Source: Immunohorizons. 2024 Sep 16;8(9):695–711. doi: 10.4049/immunohorizons.2400053 (PMC11447685; doi:10.4049/immunohorizons.2400053)
Supplement: Supplemental Material (PDF) [file IH_2400053_Supplemental_1.pdf]

Supplemental Fig. 1

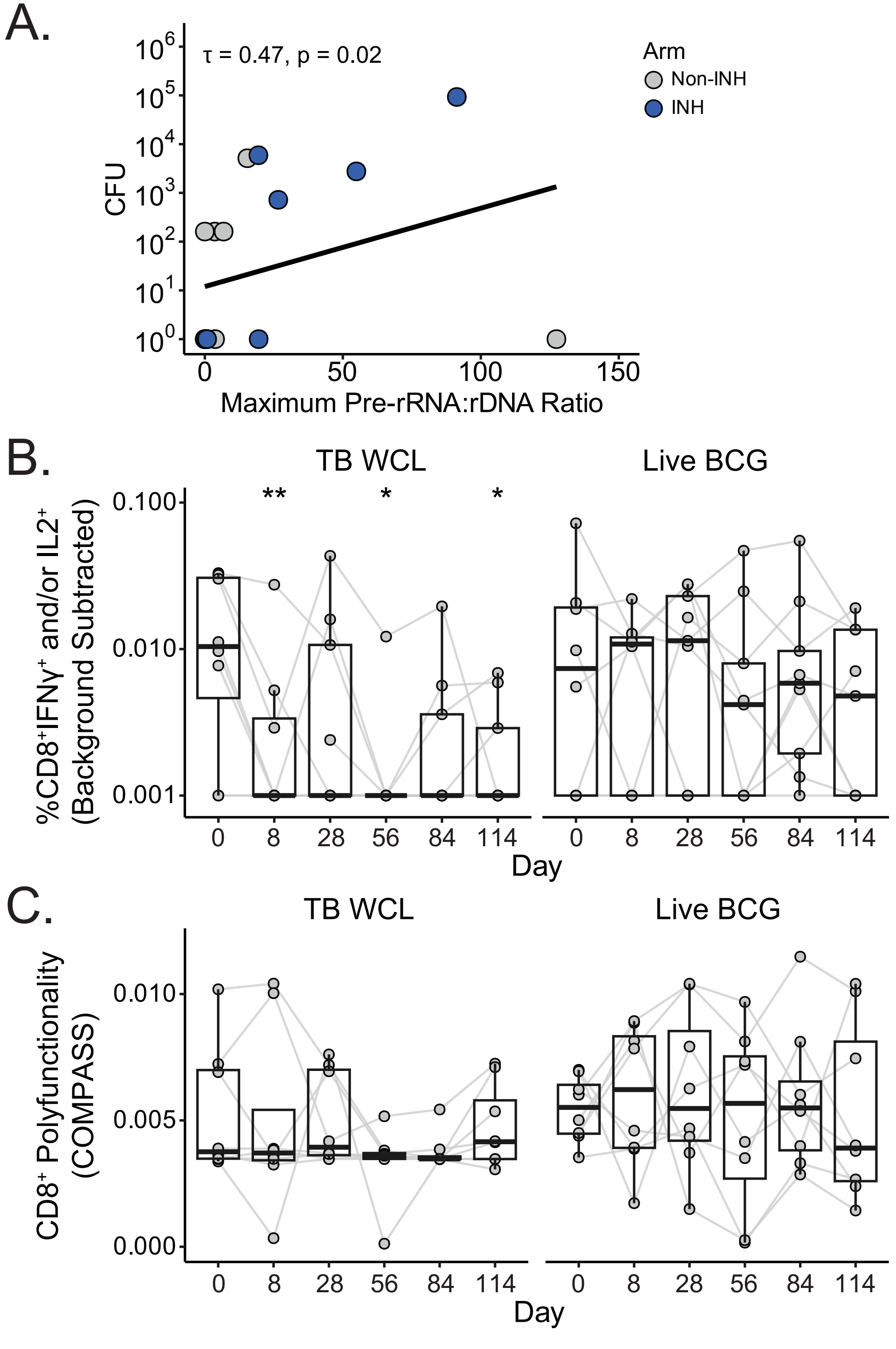

Supplemental Figure 1. Correlation of microbiological measurements and peripheral CD8+ T cell responses to Mtb or BCG stimulation. (A) CFU correlates well with maximum pre-rRNA:rDNA ratio (MVT) by Kendall correlation (INH = 10; Non-INH = 8). (B-C) Flow cytometry CD8+ T cell intracellular cytokine staining (ICS), and COMbinatorial Polyfunctionality analysis of Antigen-Specific T cell Subsets (COMPASS) analysis results (n = 8 days 0, 8, and day 114 TB WCL; n = 9 days 28, 56, 84, and day 114 BCG TICE) (B) Proportion of CD8+ T cells out of total CD45+ cells expressing IFN-γ and/or IL-2 as measured by ICS after stimulation with *Mtb* whole-cell lysate (TB WCL) or live BCG. (C) COMPASS polyfunctionality scores (PFS) after stimulation with TB WCL or live BCG. PFS reflects the proportion of cells expressing a range of cytokines, weighted by the number of cytokines expressed per cell. Statistical significance for (B) and (C) was calculated using paired Wilcoxon signed rank tests. *Unadjusted p-value <0.05, **unadjusted p-value <0.01

Supplemental Fig. 2

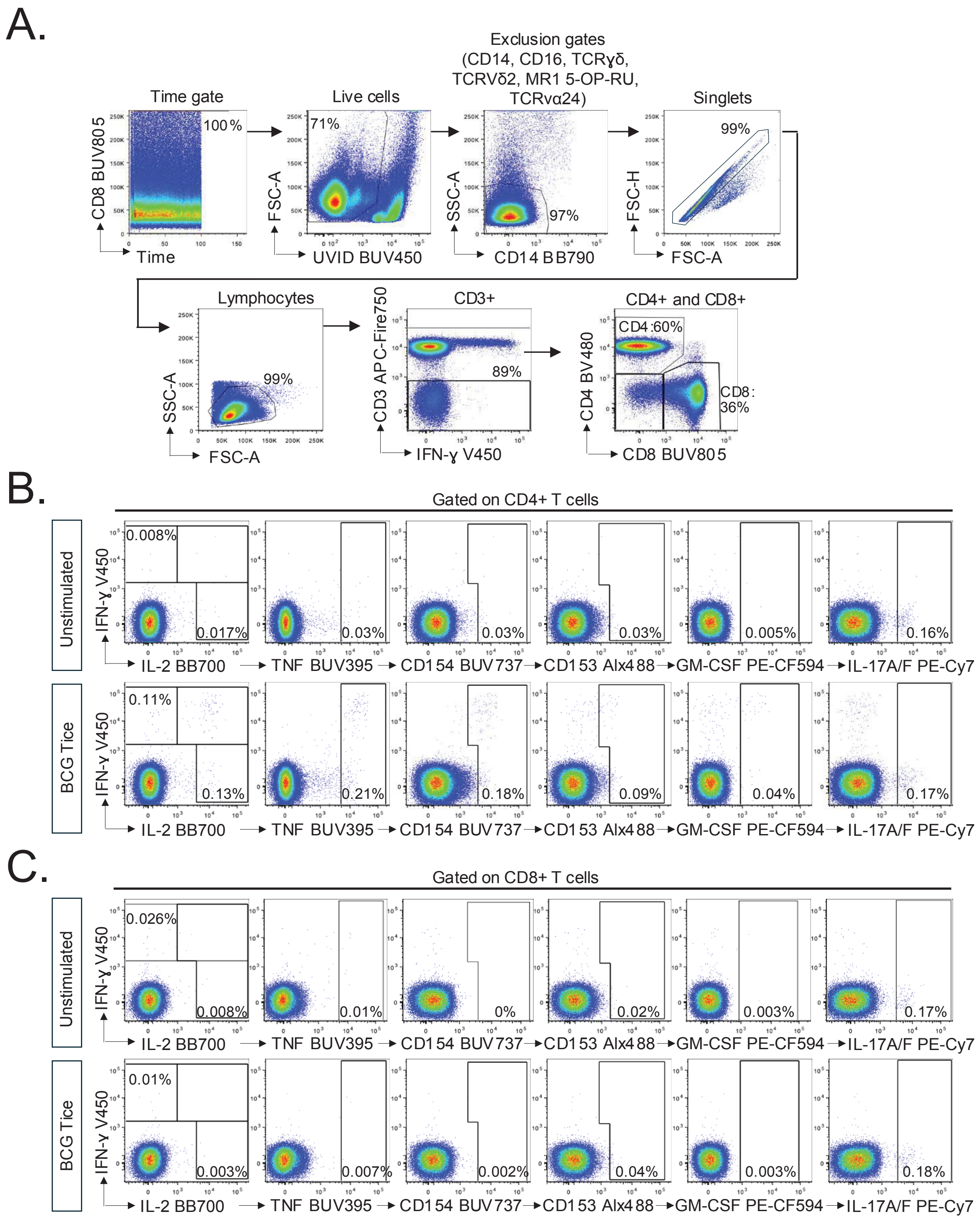

Supplemental Figure 2. Gating strategy for intracellular cytokine staining (ICS) performed on cryopreserved PBMC. (A) Nested gating strategy to identify CD4+ and CD8+ T cell populations. (B-C) Expression of IFN-γ, IL-2, TNF, CD154 (CD40L), CD153, GM-CSF, IL17A and IL-17F in (B) CD4+ and (C) CD8+ T cells in Unstimulated cells and after stimulation with BCG Tice.

Supplemental Fig. 3

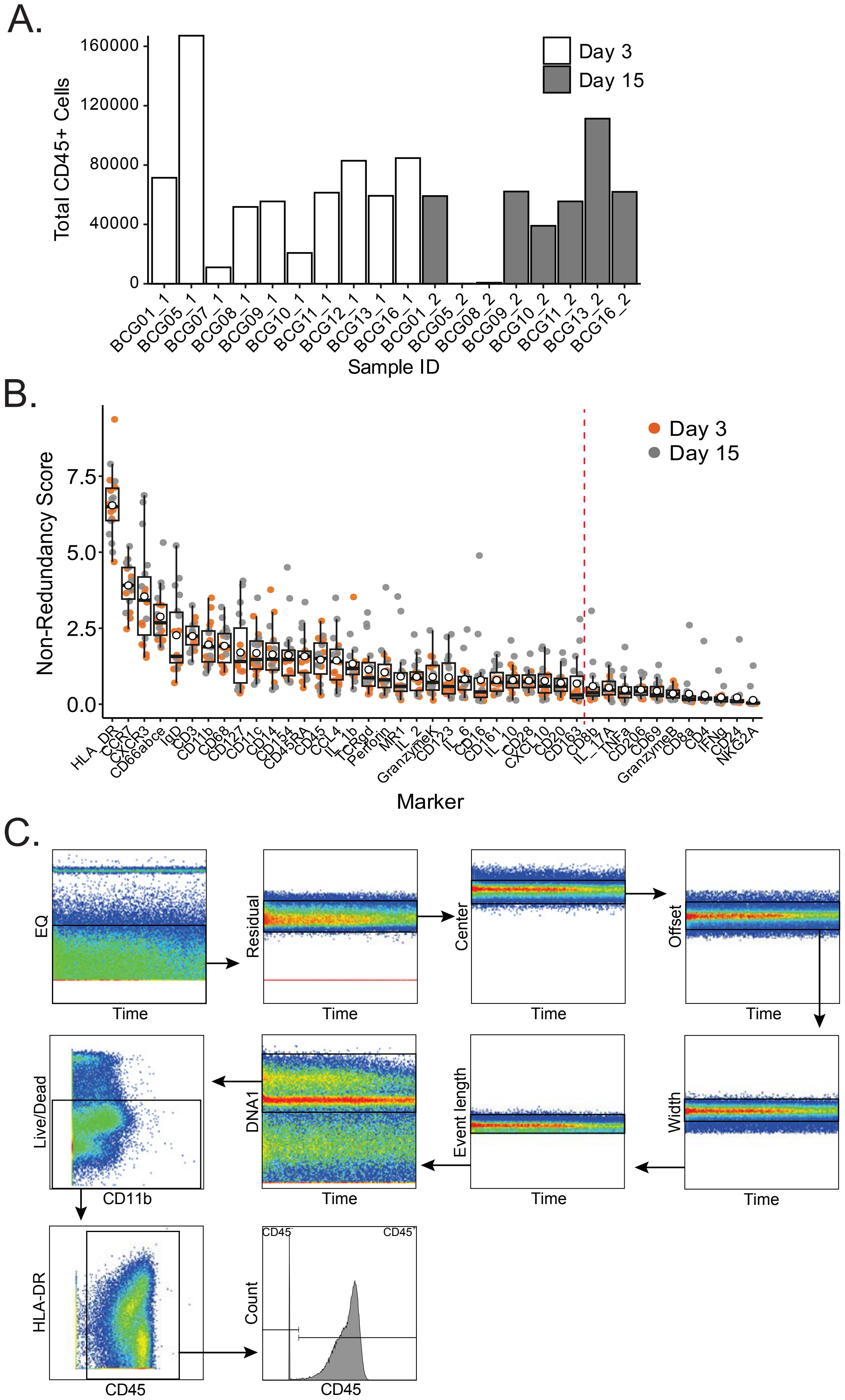

Supplemental Figure 3. CyTOF quality control and gating strategy. (A) Cell counts from each sample. There was no day 15 biopsy for participant BCG12. (B) Non-redundancy scores for each marker, calculated using the CATALYST R package (day 3 n = 10; day 15 n = 8). The dashed red line delineates the eleven lowest-ranking markers, which include the seven surface markers that were excluded from analysis. (C) Representative gating tree which was used in FlowJo 10 before exporting and analyzing CD45+ cell data in R.

Supplemental Fig. 4

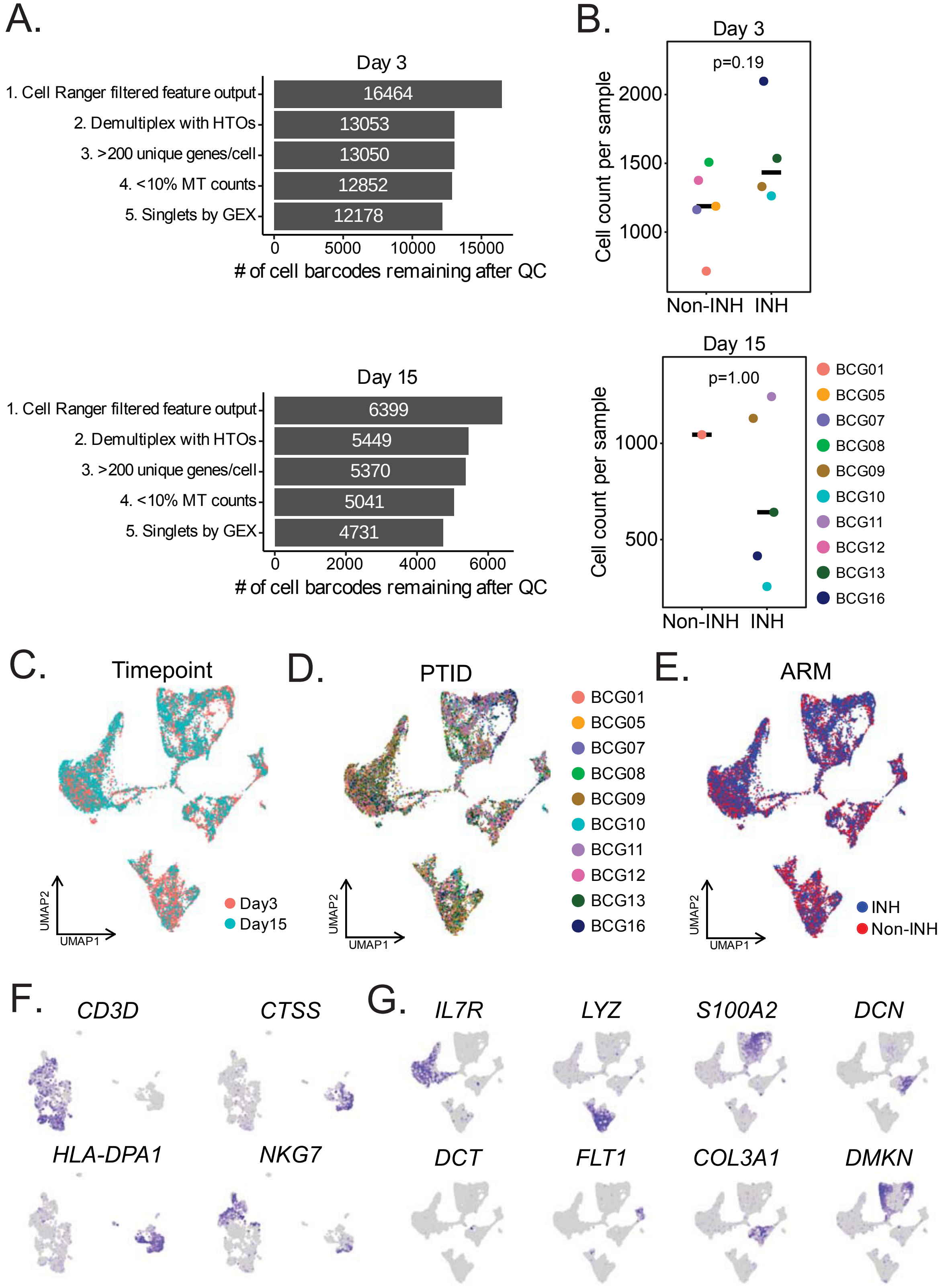

Supplemental Figure 4. scRNA-seq quality control. (A) Cell counts across five mRNA pre-processing steps. First, the mRNA count matrices were obtained from the filtered Cell Ranger multi pipeline output. Next, data were demultiplexed based on hashtag oligo (HTO) enrichment, and singlet cell barcodes with >200 unique genes/cell and <10% mitochondrial counts were retained. Lastly, the scDblFinder package was used to exclude within-sample doublets using gene expression. (B) Cell counts for all samples passing the mRNA preprocessing steps outlined in A. For (A) and (B) day 3 n = 9; day 15 n = 6. (C-E) UMAP visualizations of integrated scRNA-seq data of all cells passing the pre-processing steps outlined in (A) and annotation quality control (n = 15). Cells colored by timepoint (C), participant (D), and treatment arm (E). (F-G) Feature plots depicting the gene expression pattern of immune (F) and major (G) cell population markers (n = 15). CD3D, T cell; CTSS, monocyte; HLA-DPA1, dendritic cell; NKG7, NK cell; IL7R, lymphoid; LYZ, myeloid; S100A2, epithelial; DCN, connective tissue; DCT, nervous tissue; FLT1, endothelial; COL3A1, muscle tissue; DMKN, keratinocyte.

Supplemental Table I. Inclusion and exclusion criteria. A total of 10 participants were enrolled. This study is registered at Clinicaltrials.gov (NCT05592223).

Inclusion Criteria

Provide written informed consent prior to initiation of any study procedures.
Are males or non-pregnant females between the ages of 18 and 45 years, inclusive.
Women of childbearing potential* in sexual relationships with men must use an acceptable method of preventing conception** from 30 days prior to 3 months after Tice® BCG administration.
For women of childbearing potential, negative serum pregnancy test at screening and negative urine pregnancy test within 24 hours prior to enrollment and Tice® BCG
Are in good health, as judged by the investigator and determined by vital signs (oral temperature, pulse, and blood pressure), medical history and physical examination.
Have a negative HIV-1 ELISA test.
Have negative serology tests for hepatitis B surface antigen and hepatitis C virus antibody.
Have a negative QuantiFERON-TB Gold test (Negative is defined as Nil response < 0.8 IU/ml and TB Antigen response minus Nil response <0.35 IU/mL or TB Antigen response minus Nil response > 0.35 IU/mL and < 25% of Nil response and Mitogen response minus Nil response > 0.5 IU/ml).
Have a urine dipstick for protein less than 1.
Have a urine dipstick negative for glucose.
Ability to understand and complete all study visits as required per protocol and be reachable by telephone.

Exclusion Criteria

Have a history of suspected, confirmed, treated or have other evidence of active tuberculosis.
Have any systemic symptoms within 72 hours before Tice® BCG administration or signs of lymphadenopathy, hepatosplenomegaly, or pulmonary disease by physical examination on day of Tice® BCG administration.
Have history of any significant acute or chronic medical conditions or need for chronic medications that, in the opinion of the investigator, will interfere with immunity or
Have any history of excessive scarring or keloid formation.
Have household contact or occupation involving significant contact with someone who is immunocompromised (Includes persons with HIV, AIDs, or active cancer; infants < 1 year; pregnant women; or persons who are immunosuppressed for approximately 6 weeks during the time of active ID lesion drainage).
Have a history of epilepsy (does not include febrile seizures as a child).
Have a pacemaker, prosthetic valve, or implantable cardiac devices.
Have a history of bleeding disorder.
Have a known allergy to any Tice® BCG components (glycerin, asparagine, citric acid, potassium phosphate, magnesium sulfate, iron ammonium citrate, and lactose).
Received blood products or immunoglobulin within 6 months prior to Tice® BCG administration.
Received immunotherapy within one year prior to Tice® BCG administration.
Received or plan to receive live attenuated vaccines 4 weeks before or after Tice® BCG administration.
Received or plan to receive inactivated or killed vaccines 2 weeks before or after Tice® BCG administration.
Plans to enroll in another clinical trial with an active intervention that could interfere with safety assessment of the investigational product at any time during the study
Received an experimental agent within 30 days prior to Tice® BCG administration or planned receipt of an experimental agent within 90 days after Tice® BCG administration (Includes vaccine, drug, biologic, device, blood product, or medication).
Have a history of use of a systemic antibiotic within 14 days prior to Tice® BCG administration or planned use of a systemic antibiotic for 3 months after Tice® BCG
Have any medical, psychiatric, occupational, or behavioral problems that make it unlikely for the subject to comply with the protocol as determined by the investigator.
Are health care providers at the highest risk of acquiring MTB infection, such as pulmonologists performing bronchoscopies on TB patients.
Are breastfeeding or plan to breastfeed at any given time throughout the study.
Have long term use [ǂ] of high dose oral or parenteral glucocorticoids [ǂǂ], or high-dose inhaled steroids.[ǂǂǂ]
Have immunosuppression or are taking systemic immunosuppressants as a result of an underlying illness or treatment.
Use of anticancer chemotherapy or radiation therapy (cytotoxic) within 36 months prior to Tice® BCG administration.
Any active neoplastic disease.
Have a pulse rate less than 50 bpm or greater than 100 bpm.
Have a systolic blood pressure less than 90 mm Hg or greater than 140 mm Hg.
Have a diastolic blood pressure less than 50 mm Hg or greater than 90 mmHg.
Have a WBC less than 4.0x10^3/uL or greater than 10.5x10^3/uL.
Have hemoglobin less than 11.5x10^3/uL (female) or less than 12.5x10^3/uL (male).
Have a platelet count less than 140x10^3/UL.
Have a creatinine greater than 1.30 mg/dL.
Have an ALT (SGPT) greater than 40 IU/L (female) or greater than 55 IU/L (male).
Have known HIV, Hepatitis B, or Hepatitis C infection.
Have a history of alcohol or drug abuse in the last 5 years.
Have had a positive PPD skin test in the past or received BCG vaccine (BCG vaccination history will be determined by self-report, country of birth, and/or evidence of BCG
Have a BMI >35.
PPD skin test within 2 months prior to Tice® BCG administration or planned receipt during the study other than from participation in this study.
Oral temperature ≥ 100.4°F (≥ 38.0°C) or other symptoms of an acute illness within 3 days before Tice® BCG administration (Subject may be rescheduled).
Any medical disease or condition that, in the opinion of the investigator, is a contraindication to study participation.

* Not sterilized via tubal ligation, bilateral oophorectomy, hysterectomy or successful Essure® placement (permanent, non-surgical, non-hormonal sterilization) with documented radiological confirmation test at least 90 days after the procedure, and still menstruating or < 1 year of the last menses if menopausal).
** Includes, but is not limited to, sexual abstinence, monogamous relationship with vasectomized partner who has been vasectomized for 6 months or more prior to the subject receiving Tice®BCG, barrier methods such as condoms or diaphragms with spermicide or foam, effective intrauterine devices, NuvaRing ®, and licensed hormonal methods such as implants, injectablesor oral contraceptives (“the pill”).
[ǂ] Defined as taken for 2 weeks or more in total at any time during the past 2 months.
[ǂǂ] High dose defined as prednisone ≥ 20 mg total daily dose, or equivalent dose of other glucocorticoids.
[ǂǂǂ] High dose defined as > 800 mcg/day of beclomethasone dipropionate or equivalent. If short term corticosteroids are given, then the subject should not receive Tice® BCG or have blood collected for immunogenicity studies within 1 week of steroid administration.
